# A New Sugar for an Old Phage: a c-di-GMP-Dependent Polysaccharide Pathway Sensitizes *Escherichia coli* for Bacteriophage Infection

**DOI:** 10.1128/mbio.03246-21

**Published:** 2021-12-14

**Authors:** Benjamin Sellner, Rūta Prakapaitė, Margo van Berkum, Matthias Heinemann, Alexander Harms, Urs Jenal

**Affiliations:** a Biozentrum of the University of Basel, Basel, Switzerland; Institut Pasteur

**Keywords:** *Escherichia coli*, phage receptor, phage infection, N4 phage, NfrA, NfrB, c-di-GMP, allosteric activation, WecB, ECA, ManNAc, glycosyltransferase, exopolysaccharide, dCache domain, arginine, PdeL, DgcJ, DgcQ

## Abstract

Bacteriophages are ubiquitous parasites of bacteria and major drivers of bacterial ecology and evolution. Despite an ever-growing interest in their biotechnological and therapeutic applications, detailed knowledge of the molecular mechanisms underlying phage-host interactions remains scarce. Here, we show that bacteriophage N4 exploits a novel surface glycan (NGR) as a receptor to infect its host Escherichia coli. We demonstrate that this process is regulated by the second messenger c-di-GMP and that N4 infection is specifically stimulated by the diguanylate cyclase DgcJ, while the phosphodiesterase PdeL effectively protects E. coli from N4-mediated killing. PdeL-mediated protection requires its catalytic activity to reduce c-di-GMP and includes a secondary role as a transcriptional repressor. We demonstrate that PdeL binds to and represses the promoter of the *wec* operon, which encodes components of the enterobacterial common antigen (ECA) exopolysaccharide pathway. However, only the acetylglucosamine epimerase WecB but none of the other ECA components is required for N4 infection. Based on this, we postulate that NGR is an *N*-acetylmannosamine-based carbohydrate polymer that is produced and exported to the cell surface of E. coli in a c-di-GMP-dependent manner, where it serves as a receptor for N4. This novel carbohydrate pathway is conserved in E. coli and other bacterial pathogens, serves as the primary receptor for various bacteriophages, and is induced at elevated temperature and by specific amino acid-based nutrients. These studies provide an entry point into understanding how bacteria use specific regulatory mechanisms to balance costs and benefits of highly conserved surface structures.

## INTRODUCTION

Bacteriophages are ubiquitous predators of their bacterial hosts and drive their ecology and evolution in a tight arms race (1). The host range of bacteriophages is predetermined by the recognition of specific receptors on the bacterial cell surface using receptor-binding proteins that are displayed by tailed phages on their tail fibers, tailspikes, or similar structures (2). While exposed glycan structures are often used as a first “primary” receptor for host recognition, irreversible adsorption and injection of the phage genome are triggered by subsequent binding to a terminal or “secondary” receptor directly on the cell surface (2). For Gram-negative bacteria like the model organism Escherichia coli, all known types of glycans, including capsules, the highly variable O‐antigen chains of lipopolysaccharide (LPS), and the conserved yet enigmatic enterobacterial common antigen (ECA) have been described as primary receptors for phage docking (3, 4–7). However, surface-exposed polysaccharides also play major roles in bacterial defense against phages since they can shield terminal receptors on the cell surface (4, 5, 8, 9). For example, in E. coli K-12 O-antigen expression was shown to eliminate the adsorption of a wide range of bacteriophages that could bind diverse terminal receptors and infect productively in the absence of this barrier (3). Likewise, overproduction of capsules can effectively protect bacteria from phage adsorption (10, 11). This dual role of surface glycans as barrier and receptor is mirrored on the phage side in form of tailspikes. These are tail fibers decorated with glycan-targeting enzymes that specifically recognize certain sugar motifs on host exopolysaccharides and then modify or degrade them unit by unit to drive translocation of the virion along the polysaccharide chain toward the cell surface (12–14).

Previous genetic studies indicated that the ECA glycan chains are exploited as host receptor by diverse and very common bacteriophages like the well-studied podovirus N4 (3, 15). This is remarkable since—unlike the almost 200 different types of O antigens for E. coli alone (16)—the ECA glycan is invariable across enterobacteria, possibly due to functional constraints in their interaction with their animal hosts (17). To ease the selective pressure imposed by phage predation via conserved surface structures such as ECA, bacteria have evolved different strategies, including tightly regulating such surface components (18–20). Understanding how bacteria maintain the expression of highly conserved surface glycans, despite phage predation, is not only relevant for phage ecology and evolution but could also have great value for the therapeutic use of bacteriophages. Consequently, the *bona fide* ECA-targeting phages studied in previous work systematically displayed the broadest host recognition of all phages tested (3) which is, intuitively, a key property when selecting phages for therapeutic purposes (21).

**FIG 1:**
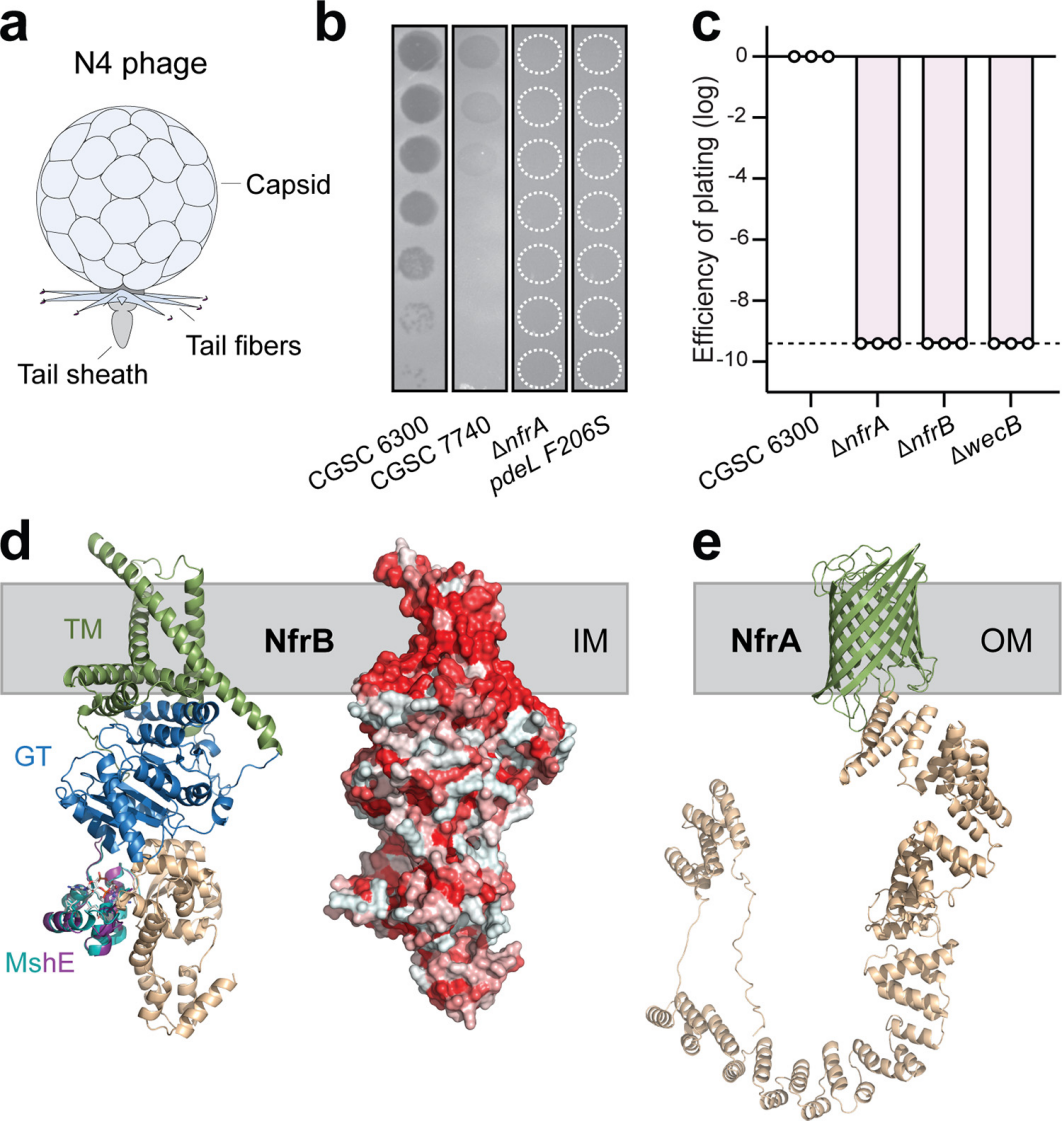
Infection of E. coli by bacteriophage N4 requires components of a putative surface glycan secretion system. (a) Schematic of bacteriophage N4. (b) Plaque assay with serial 10-fold dilutions of bacteriophage N4 spotted on lawns of different E. coli host strains as indicated. Stippled white circles indicate regions of phage application where no lysis was observed. (c) The efficiency of plating (EOP) is displayed for several E. coli host strains as the number of PFU relative to E. coli wild-type strain CGSC6300. All mutants are in an CGSC 6300 background. Circles indicate the average of two technical replicates of one biological repeat, and the bar indicates the mean of the log-transformed EOP values. The stippled line marks the detection limit. (d) Model of the structure of NfrB as predicted by AlphaFold (30). (Left) Colored domains of NfrB with homology to glycosyltransferases (GT, blue), the c-di-GMP binding domain (cyan, purple), and a domain with unknown function (sand). Putative transmembrane helices (TM) incorporated in the inner membrane (IM) are indicated in green. The putative c-di-GMP binding domain of NfrB (purple) is shown as overlap with the c-di-GMP binding domain of the MshE ATPase from V. cholerae (33) with bound ligand (teal). (Right) Depiction of the NfrB surface with hydrophobic amino acids indicated in red. (e) Structural model of NfrA as predicted by AlphaFold (30). The outer membrane (OM) beta-barrel structure is indicated in green, and the TPR domains and unstructured regions are shown in sand.

In this study, we explored the molecular basis of host recognition by the podovirus N4 (Fig. 1a), a member of the *Schitoviridae* that infect E. coli (22). Selection for mutations conferring N4 resistance (*nfr*) had uncovered genes *nfrA*, *nfrB*, and *nfrC* as candidates for phage entry (15, 23). The *nfrA* gene encodes an outer membrane protein that was described to interact with the tail sheath protein of N4 and might be the terminal receptor for N4 (24). The *nfrC* allele was mapped to *wecB*, a gene encoding a cytoplasmic UDP-*N*-acetylglucosamine 2-epimerase that is part of a large gene cluster involved in the synthesis of ECA (25). Because ECA is the only known exopolysaccharide that depends on WecB, it was proposed that N4 uses ECA as its primary surface receptor to infect E. coli (23, 26). Apart from its requirement for N4 and related phages in the *Enquatrovirus* genus of *Schitoviridae*, WecB was recently also shown to be required for infectivity of myoviruses of the *Vequintavirinae* subfamily and their phi92-like relatives (3). Intriguingly, all of these phages encode homologous glycan deacetylase tailspikes, indicating that they target surface glycans in a similar way.

Our results show that bacteriophage N4 and other phages previously linked to ECA do not target the ECA as their primary receptor but instead use a novel surface glycan of E. coli that we call NGR (N4 Glycan Receptor). We present evidence that NGR is produced and exported by a conserved biosynthesis machinery, including WecB, NfrA, and NfrB. Similar to ECA components, the genes encoding this machinery are widespread among enterobacteria and some related groups, providing an elegant explanation for the unusually broad host recognition of N4-like phages. Furthermore, we show that N4 infectivity critically depends on the second messenger c-di-GMP and that this requires the catalytic activity of a single diguanylate cyclase, DgcJ, possibly via a direct and local activation of the NfrB glycosyltransferase. An accompanying study strengthens this view by demonstrating that NfrB indeed binds c-di-GMP and that DgcJ directly interacts with the presumable glycosyltransferase (27). Thus, our study not only sheds new light on the molecular mechanisms underlying bacteriophage host range but also provides an entry point into understanding how bacteria use local signaling via the second-messenger c-di-GMP to balance costs and benefits of surface glycan expression.

## RESULTS

### Infection of *E. coli* by phage N4 depends on a putative exopolysaccharide pathway.

To analyze the requirements for N4 infection, we first confirmed that chromosomal deletions of the known N4 resistance genes *nfrA*, *nfrB*, and *nfrC* (*wecB*) effectively protect E. coli from N4 infection (Fig. 1b and c) (15, 23, 26). Since *nfr* mutants were shown to prevent phage adsorption (15), their products could either directly serve as receptors or could be involved in the production of surface exposed structures that are N4 receptors. Using structure-based protein comparison (28, 29) and neural network-based structure prediction (30) tools, we identified NfrA and NfrB as potential components of a novel exopolysaccharide secretion system. The N-terminal domain of NfrB shows strong homology to glycosyltransferases such as the cellulose synthase BcsA (31) (Fig. 1d), while the C terminus contains a domain of unknown function and a small MshEN-like domain, a c-di-GMP binding module involved in regulating diverse motor ATPases of type IV pili and type 2 secretion systems (32, 33) (Fig. 1d). The modeled structure of NfrA shows strong homology to exopolysaccharide translocation pores located in the outer membrane of E. coli or P. aeruginosa, including PgaA (34), BcsC (35), or AlgE (36). The NfrA N terminus contains several tetratricopeptide repeat (TPR) units, which in other glycan translocation pores were hypothesized to interact with periplasmic polymer-modifying enzymes or with the synthase complex located in the inner membrane (35). The C terminus of NfrA is a 16-stranded β barrel pore with structural similarities to other glycan translocation pores, including PgaA (34) or BcsC (35) (Fig. 1e; see also Fig. S1). Based on this, we postulate that NfrB and NfrA are part of a multicomponent glycan synthase complex and that their strict requirement for N4 infection may indicate the existence of a novel E. coli exopolysaccharide that serves as the primary receptor for N4. Based on this assumption, we term this unknown exopolysaccharide N4 glycan receptor (NGR), and we use N4 infection assays from here on to probe the regulation of the Nfr-mediated NGR biogenesis/secretion.

10.1128/mbio.03246-21.1 FIG S1 Structural similarities indicate a functional link between NfrA, BcsC, and PgaA. Structural predictions using AlphaFold (30) were performed on NfrA, BcsC, and PgaA. The C-terminal outer membrane beta-barrel structure is indicated in green, and TPR domains and unstructured regions are shown in sand. Download FIG S1, PDF file, 2.8 MB. Copyright © 2021 Sellner et al. 2021 Sellner et al. https://creativecommons.org/licenses/by/4.0/ This content is distributed under the terms of the Creative Commons Attribution 4.0 International license.

### N4 infection requires c-di-GMP.

**FIG 2:**
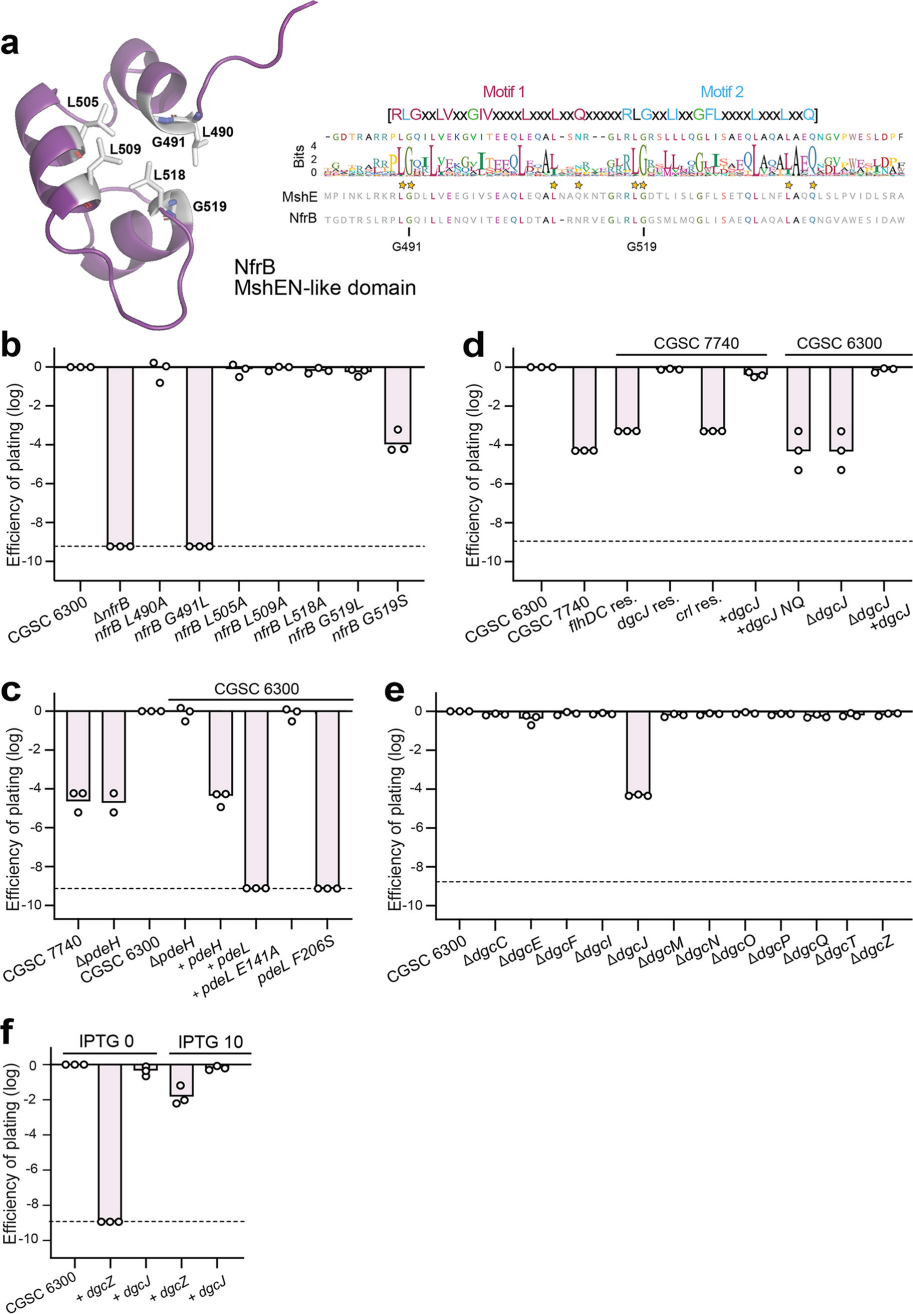
c-di-GMP is required for N4 infection. (a) NfrB harbors a domain with strong homology to a c-di-GMP binding domain of V. cholerae MshEN. (Left) Close-up of a structural model of the putative c-di-GMP binding domain of NfrB (purple). Conserved residues of the c-di-GMP binding pocket (33) that were used for the mutational analysis in panel b are indicated in sticks. (Right) ClustalW sequence alignment of the 1,000 closest homologues of E. coli NfrB using all *Enterobacterales* but excluding the genus E. coli. The conservation of the putative c-di-GMP binding motif of NfrB homologs is shown by the sequence logo with residues of MshEN involved in c-di-GMP binding marked by stars. The amino acid sequences of MshEN and NfrB are shown below the logo. (b) Conserved residues of the MshEN-like domain of NfrB are required for N4 infection. Circles indicate the EOP with N4 phages; displayed for E. coli wild-type and mutant strains as described in Fig. 1. The stippled line marks the detection limit. (c) The phosphodiesterase PdeL effectively protects E. coli against N4 infection. EOP is displayed for E. coli wild-type (strain CGSC 6300 or CGSC 7740) and mutant strains. Strains harboring a plasmid-born copy of *pdeL* wild type, *pdeL* mutant alleles, or *pdeH* transcribed from an IPTG-inducible promoter (P_lac_) are indicated. (d) An IS*1* insertion in *dgcJ* is responsible for N4 resistance of E. coli strain CGSC 7740. The EOP of phage N4 is displayed for strains CGSC 6300 and CGSC 7740 and for strains with restored wild-type sequences (res.) at the chromosomal loci *flhDC*, *crl*, and *dgcJ*, as indicated. Strains containing chromosomal deletions of *dgcJ* and strains carrying *dgcJ* alleles on a plasmid (+) are indicated, whereas *dgcJ* NQ indicates a catalytically inactive *dgcJ* allele. (e) Specific requirement of the DgcJ diguanylate cyclase for N4 phage infection. The EOP of phage N4 is displayed for E. coli wild-type and deletion strains lacking individual diguanylate cyclases. (f) Ectopic expression of *dgcJ* sensitizes E. coli toward N4 infection in a *dgcJ dgcQ* knockout strain. Expression of the DGC *dgcJ* or *dgcZ* in a *dgcJ/dgcQ* double knockout strain was induced from the lactose promoter with 0 or 10 μM IPTG.

The presence of a MshEN-like domain—typically mediating the allosteric regulation of proteins by c-di-GMP (33, 37)—in NfrB indicated that its activity may be controlled by c-di-GMP. To test this, we first mutagenized residues that are conserved between NfrB and MshEN and that were shown to be involved in c-di-GMP binding (33) and analyzed their effect on N4 infection. This included Leu490, Gly491, Leu505, Leu509, Leu518, and Gly519 (Fig. 2a). While most substitutions showed no effect on N4 infection, G491L and G519S abolished N4-mediated killing completely and partially, respectively (Fig. 2b). This is consistent with the observed key role of Gly residues in c-di-GMP binding to the MshEN domain of Vibrio cholerae (33) and indicated that c-di-GMP binding to NfrB is required for phage infection.

Next, we investigated N4 infection in several lab adapted strains of E. coli. We found that while the original E. coli K-12 MG1655 strain (CGSC 6300) (38) was susceptible to N4, a closely related hyper-motile variant (CGSC 7740) (39) (see Fig. S2a) showed strong resistance toward N4 infection (Fig. 2c and d). Strain CGSC 7740 carries an IS*1* insertion upstream of *flhDC*, which encodes the master regulator of the flagellar regulon (40) (see Fig. S2b). Because this insertion leads to the constitutive expression of flagellar genes and the phosphodiesterase gene *pdeH*, c-di-GMP levels are substantially reduced in strain CGSC 7740 compared to strain CGSC 6300 (41). This strengthened the idea that c-di-GMP plays an important role in NGR biogenesis and argued that constitutive expression of *pdeH* may be responsible for N4 resistance of CGSC 7740. However, N4 sensitivity was not restored when deleting *pdeH* in the CGSC 7740 background (Fig. 2c) despite the fact that global c-di-GMP levels increased 10-fold and motility was strongly impaired (41). Likewise, restoring the original *flhDC* locus by removing of the IS*1* element, although effectively blocking motility (see Fig. S2b), failed to restore N4 sensitivity (Fig. 2d). Finally, expression of *pdeH* from a plasmid in strain CGSC 6300 provided only limited protection against N4 (Fig. 2c). From these experiments, we concluded that although c-di-GMP is required for N4 infection, global changes of c-di-GMP levels do not strongly influence N4-mediated killing of E. coli.

10.1128/mbio.03246-21.2 FIG S2 Identification of IS*1* elements in E. coli MG1655 CGSC 7740. (a) Motility of E. coli strains CGSC 6300 (1) and CGSC 7740 (2) scored on a motility agar plate (TB plus 0.3% agar). (b) Removing different IS*1* insertions to restore the original wild-type sequence of strain CGSC 7740. Strains with different combinations of restored wild-type loci are shown at the top, and PCR amplification of the *flhDC* promoter, and the *crl* and *dgcJ* open reading frames before (A) and after restoration (B) are shown at the bottom. On the right is a motility with different E. coli strains, as indicated on the left. (c) Motility of E. coli CGSC 7740 carrying plasmids expressing *dgcZ*, *dgcJ NQ* (catalytically inactive), *dgcJ* (wt), or empty vector as indicated. (d, left) Schematic of the chromosomal region harboring the *dgcJ* gene in strains CGSC 6300 (top) and CGSC 7740 (bottom) with the position of the IS*1* insertion marked. Filled triangle, location of IS*1* insertion; open triangle, stop codon introduced by IS*1* element insertion. Sequences in the boxes show the *dgcJ* sequence (white) with duplicated sequences of *dgcJ* (yellow) and IS*1* (gray). (Right) Schematic of DgcJ with the N-terminal dCache domain and the C-terminal GGDEF domain as indicated. Prediction and visualization were performed using PROTTER (82). Download FIG S2, PDF file, 0.5 MB. Copyright © 2021 Sellner et al. 2021 Sellner et al. https://creativecommons.org/licenses/by/4.0/ This content is distributed under the terms of the Creative Commons Attribution 4.0 International license.

### The diguanylate cyclase DgcJ regulates Nfr-dependent N4 infection in a highly specific manner.

To decipher the molecular determinants responsible for N4 resistance of strain CGSC 7740, we reexamined its chromosome sequences (U00096) and found two additional IS*1* insertions that are not present in strain CGSC 6300 (NC_000913.3). Insertions mapped to *crl*, a gene encoding an activator of the stress sigma factor RpoS (42), and to *dgcJ*, which codes for one of several diguanylate cyclases of E. coli (43) (see Fig. S2d). Replacing IS*1* elements in strain CGSC 7740 individually with the corresponding chromosomal wild-type sequences from strain CGSC 6300 (see Fig. S2b) revealed that N4 sensitivity was only reestablished upon restoring *dgcJ* but not when the *flhDC* or *crl* loci were restored (Fig. 2d).

These findings indicated that DgcJ is a main driver of sensitivity to N4 infection. In line with this, deleting *dgcJ* in strain CGSC 6300 provided strong protection against N4, while deleting any other *dgc* gene in this background showed no effect (Fig. 2e). Ectopic expression of *dgcJ* restored N4 sensitivity of both the Δ*dgcJ* mutant in the CGSC 6300 background and of strain CGSC 7740 (*dgcJ*::IS*1*). In contrast, expression of *dgcJ* (*DE425NQ*) a mutant allele encoding a catalytically inactive variant of DgcJ, failed to restore phage sensitivity in strain CGSC 7740 (Fig. 2d). Ectopic expression of *dgcJ* also restored N4 susceptibility in a *nfrB G491L* mutant background, arguing that this mutation indeed compromised c-di-GMP binding to NfrB, a phenotype that is likely compensated by increasing the levels of this highly specific diguanylate cyclase (see Fig. S3). Finally, basal level expression of *dgcJ* readily restored N4 sensitivity, while expression of *dgcZ*, a gene encoding a highly active diguanylate cyclase from E. coli (44), failed to restore N4 sensitivity in a strain lacking *dgcJ* and *dgcQ* (Fig. 2f), despite of its potent inhibition of E. coli swimming motility under the same conditions (see Fig. S2c) (45). DgcZ could, however, partially restore N4 sensitivity when its transcription was increased by the addition of IPTG **(**Fig. 2f**)**.

10.1128/mbio.03246-21.3 FIG S3 MshE-domain mutations can be rescued with *dgcJ* overexpression. The EOP of phage N4 is displayed for the parental strain CGSC 6300 and CGSC 6300 carrying mutations in *nfrB*. Ectopic expression of *dgcJ* with 1 mM IPTG restored N4 infection in a strain carrying the G491L mutation in NfrB. Download FIG S3, TIF file, 2.3 MB. Copyright © 2021 Sellner et al. 2021 Sellner et al. https://creativecommons.org/licenses/by/4.0/ This content is distributed under the terms of the Creative Commons Attribution 4.0 International license.

**FIG 3:**
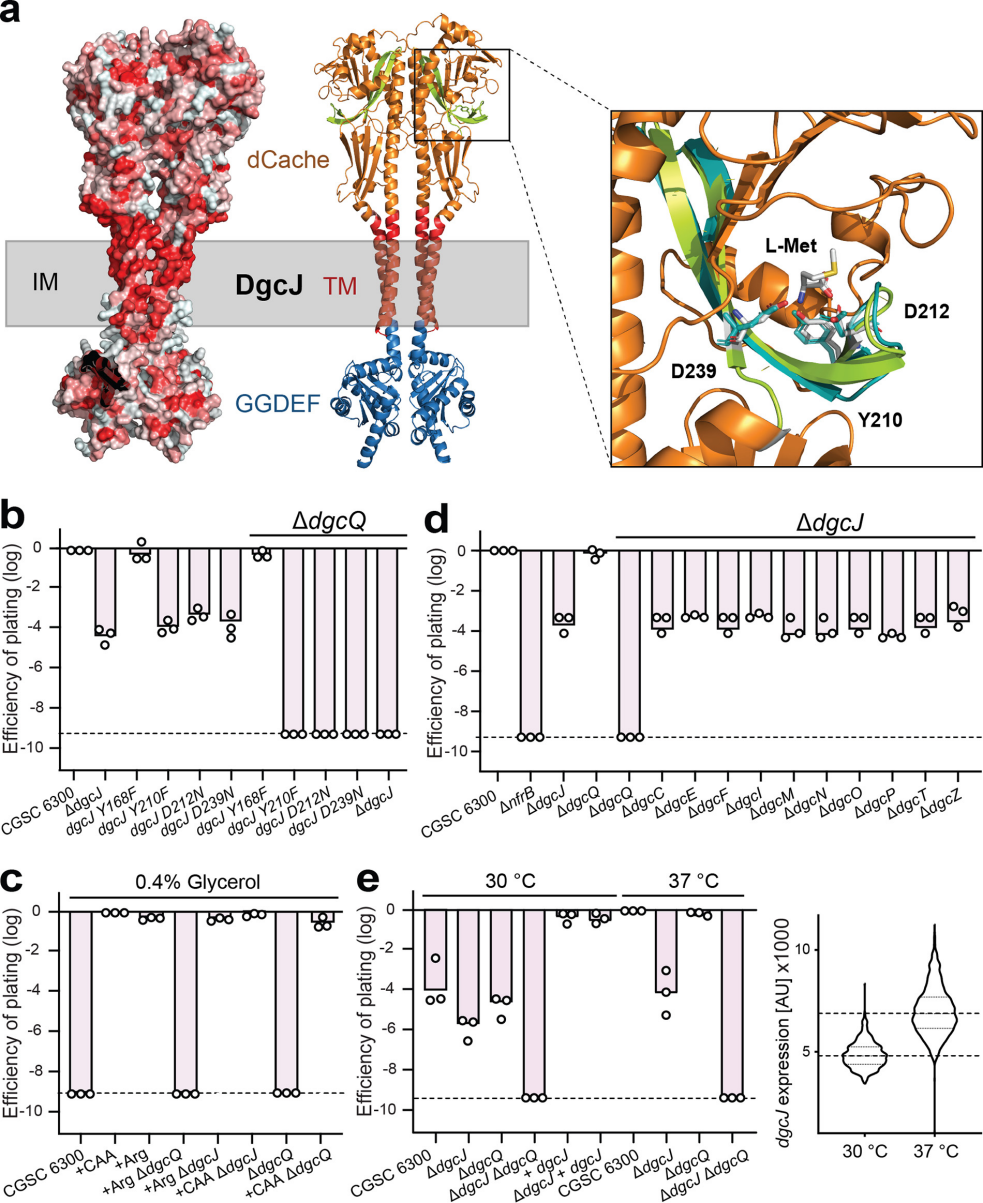
DgcJ and DgcQ, two homologous diguanylate cyclases that specifically sensitize E. coli for N4 infection. (a) Model of the dimer structure of DgcJ as predicted by AlphaFold (30). (Left) Hydrophobicity is displayed by red coloring, highlighting the TM domain. (Right) Colored domains of DgcJ with homology to dCache domain (orange) with the putative ligand-binding domain (LBD, green) and the GGDEF domain (blue). Putative TM helices incorporated in the inner membrane (IM) are indicated in red. The closeup view of the putative LBD reveals homology of the DgcJ residues Y210, D212, and D239 (displayed as sticks) to the ligand-interacting residues (teal) of PctA (47) coordinating l-methionine. (b) Isosteric mutations of putative ligand-binding residues in DgcJ phenocopy a *dgcJ* knockout. Strains carrying an additional *dgcQ* mutation are indicated above. Circles indicate the EOP with N4 phages is displayed for E. coli wild-type and mutant strains as described in Fig. 1. (c) Phage N4 infection requires extracellular amino acids. Phage infection assays were performed as described before at 37°C, but in MOPS minimal medium supplemented with 0.4% glycerol and, if indicated, with 0.4% Casamino Acids or arginine. Although no N4 infection could be observed in glycerol minimal medium, supplementation with Casamino Acids completely rescued phage susceptibility. Arginine restored N4 susceptibility in a *dgcQ*-dependent manner. (d) DgcJ specifically sensitizes E. coli for N4 infection in combination with DgcQ. Double mutants of DgcJ with other cyclases do not change N4 infection except a combination with DgcQ. The EOP was determined as in panel b. (e) N4 infection and *dgcJ* expression is reduced at lower temperatures. (Left) Phage infection assays were performed as described before, but at either 30 or 37°C, as indicated. (Right) A plasmid-borne fluorescent *dgcJ* promoter reporter was used to read out *dgcJ* expression with the microscope. The *y* axis indicates the pixel intensity from the fluorescent cells. Violin plots contain >1,000 individually quantified cells.

The above results indicated that DgcJ is a critical determinant for N4 infection of E. coli that activates NGR biogenesis in a highly specific manner. DgcJ is a membrane protein (46) with a periplasmic dCache domain and a cytoplasmic catalytic GGDEF domain (Fig. 3a; see also Fig. S2d). The dCache domain of DgcJ is closely related to the periplasmic domain of the methyl-accepting chemotaxis protein PctA from Pseudomonas aeruginosa, which was crystallized in complex with its amino acid ligands L-Met, L-Trp, and L-Ile (47). Some residues involved in ligand binding (Y121, Y144, D146, and D173) are conserved in DgcJ (Y168, Y210, D212, and D239) (Fig. 3a), indicating that DgcJ may bind similar ligand(s) via its periplasmic dCache domain. Consistent with this idea, isosteric substitutions of potential ligand-binding residues of DgcJ (Y210F, D212N, and D239N) invoked strong protection against phage N4, similar to levels observed for the Δ*dgcJ* mutant (Fig. 3b). Also, E. coli K-12 MG1655 (CGSC 6300) was resistant to phage N4 when grown on defined media containing glycerol as sole carbon source but was readily killed by N4 when grown in defined media supplemented with Casamino Acids (Fig. 3c). When testing amino acids individually, we found that the addition of arginine to minimal media restored phage infection in minimal medium (Fig. 3c). However, Arg-induced N4 killing under these conditions was not dependent on DgcJ, since supplementation of minimal glycerol media with arginine or with Casamino Acids also restored phage susceptibility in a Δ*dgcJ* mutant (Fig. 3c). From this, we concluded that arginine promotes N4 phage infection, possibly by activating a second diguanylate cyclase that promotes N4 infection specifically under these conditions.

To identify this second DGC, we generated all possible double mutant combinations lacking DgcJ and each of the other diguanylate cyclases of E. coli. This identified DgcQ as an additional diguanylate cyclase involved in N4 infection (Fig. 3d). While a *dgcJ* single mutant reduced N4 infection to intermediate levels in complex media, plaque formation was reduced below the detection limit in a Δ*dgcJ* Δ*dgcQ* double mutant, similar to mutants lacking NfrA or NfrB. Surprisingly, a *dgcQ* single mutant was fully susceptible to N4 in complex media (Fig. 3d), arguing that it has an auxiliary role in activating the NGR pathway. DgcQ is a homolog of the diguanylate cyclase STM1987 from Salmonella Typhimurium, which was shown to sense arginine (48). Consistent with this, DgcQ was strictly required and sufficient for infection of E. coli by phage N4 in minimal media supplemented with arginine (Fig. 3c). These experiments demonstrated that DgcQ can compensate the lack of DgcJ activity in minimal media in response to extracellular arginine. Importantly, the addition of Casamino Acids to minimal media also restored N4 susceptibility (Fig. 3c), suggesting that the dCache domain of DgcJ recognizes a nutritional signal that is contained in Casamino Acids.

Finally, we observed that E. coli was considerably less sensitive to N4 infections when grown at 30°C compared to 37°C (Fig. 3e). Ectopic expression of *dgcJ* fully restored sensitivity to phage N4 at 30°C, arguing that DgcJ levels may be limiting at 30°C. In line with this, *dgcJ* transcription was significantly reduced at 30°C compared to 37°C (Fig. 3e). Together, these results demonstrate that DgcJ expression and activity are stimulated by elevated temperatures and sensing of yet unknown ligands.

### The PdeL phosphodiesterase efficiently protects *E. coli* against N4 phage infection.

From the experiments above we concluded that c-di-GMP binds to NfrB to stimulate secretion of the NGR exopolysaccharide and that this process is regulated by the diguanylate cyclases DgcJ in a highly specific manner. The reduction of the global c-di-GMP pool through the constitutive expression of the phosphodiesterase PdeH did not effectively protect E. coli from N4 infections. Surprisingly, we found that expression of the phosphodiesterase gene *pdeL* from a plasmid phenocopied the N4 protection level observed for the Δ*nfrB* mutant (Fig. 2c). In contrast, expression of *pdeL E141A* encoding a catalytic inactive variant had no protective effect (Fig. 2c). Moreover, replacing the chromosomal wild-type copy of *pdeL* with the *pdeL* allele F206S encoding a constitutively active PdeL variant (41), provided complete protection against N4, similar to mutants lacking NfrA or NfrB (Fig. 1b and 2c).

**FIG 4:**
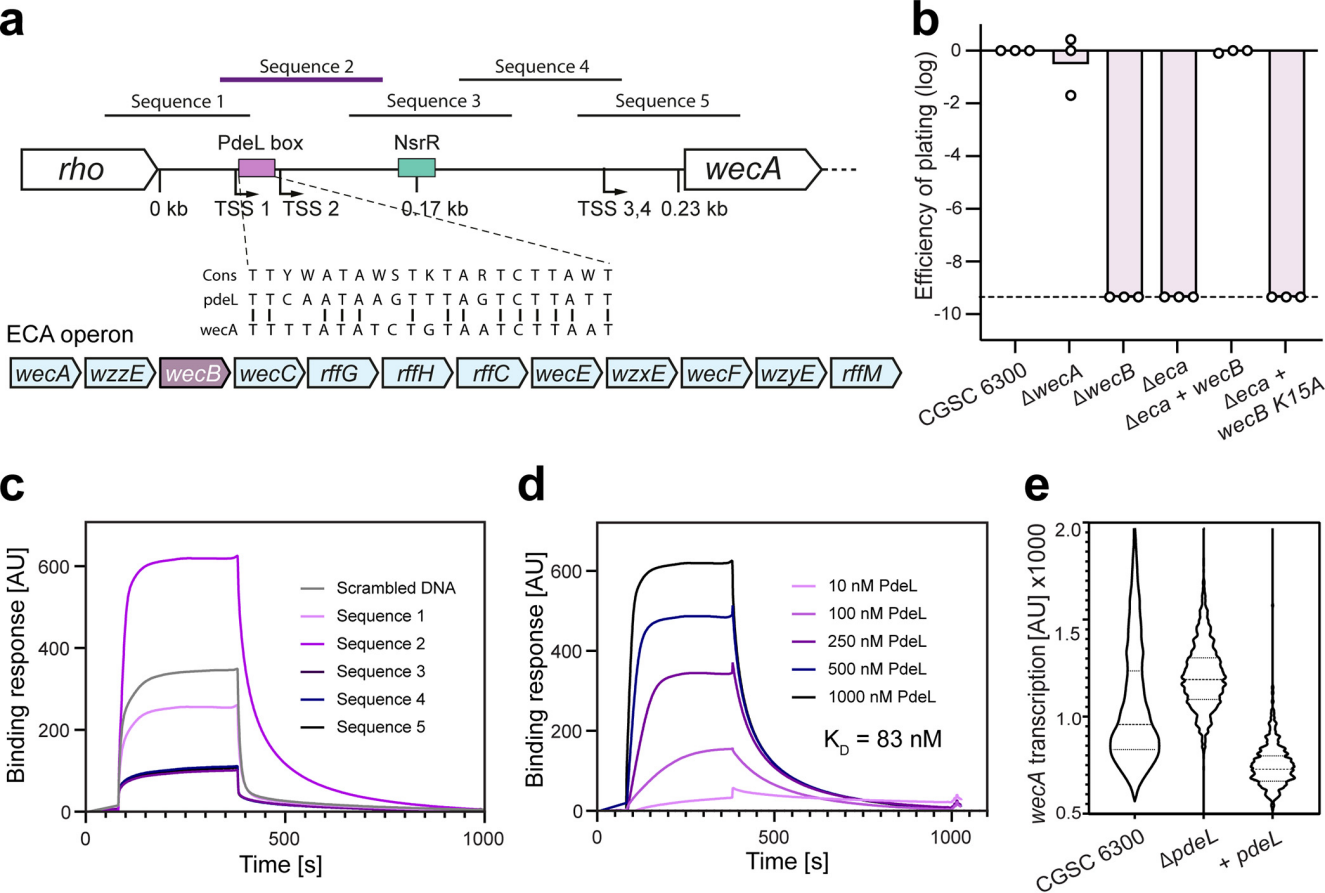
PdeL is a transcriptional repressor of the ECA operon. (a) Schematic of the *wecA* promoter region and the entire ECA operon. Transcriptional start sites (TSS) are based on (50). The binding sites of the NsrR repressor and PdeL are indicated in green and purple, respectively. The sequence of the putative PdeL binding site and homology to the PdeL binding site in the *pdeL* promoter region (41) are indicated. Sequences 1 to 5 mark the five DNA fragments used for *in vitro* binding studies using PdeL. The position of the *wecB* gene in the ECA cluster is highlighted in purple. (b) The *wecB* gene is the only gene of the ECA cluster required for N4 infection of E. coli. The EOP is displayed for E. coli wild type (CGSC 6300) and mutants with deletions in *wecA*, *wecB*, or the entire ECA gene cluster (*eca*), as described in Fig. 1. Plasmid-born copies of *wecB* alleles used for complementation of the Δ*eca* strain are indicated. Expression was induced with 1 mM IPTG. (c) PdeL binding to different fragments of the *wecA* promoter region as determined by SPR. DNA fragments (see: a) were individually immobilized on SPR chips and purified PdeL (1 μM) flushed through the flow cell. The amount of bound PdeL is shown on the *y* axis. PdeL was added 60 s after the start of recording and, after 420 s, the flow cell was flushed with buffer, resulting in PdeL dissociation from the DNA. Sequence 2 showed the strongest binding and the slowest dissociation, indicating the formation of a stable DNA-protein complex. (d) PdeL binds to the *wecA* promoter with high affinity. Experiments as outlined in panel c were carried out with immobilized DNA sequence 2 and with increasing concentrations of PdeL. The maximal binding response at ∼300 s was used to calculate the binding affinity. (e) PdeL represses the ECA operon. The activity of the *wecA* promoter was determined with a strain carrying a transcriptional *mCherry* reporter downstream of *wecA* on the chromosome. Violin plots show fluorescence distribution of at least 3,000 individual cells of E. coli wild-type and mutant strains, as indicated.

The observation that PdeL, but not PdeH, is able to effectively protect E. coli against N4 led us to investigate the molecular details of PdeL specificity. We have shown previously that PdeL is both an active phosphodiesterase and a c-di-GMP dependent transcription factor that autoregulates its own expression (41). We thus hypothesized that PdeL influences N4 infection through a combination of effectively lowering c-di-GMP levels and regulating the transcription of genes involved in N4 infection. To define additional promoters regulated by PdeL, we performed ChIP-Seq experiments using a strain expressing hemagglutinin-tagged PdeL from the chromosome. These experiments not only confirmed that PdeL binds to the *pdeL* promoter region, but also identified eight additional binding sites that were mapped to the promoter regions of *cstA*, *fruB*, *xanP*/*gltS*, *sufA*, *wecA*, *yafC/yafD*, *sslE*, and *yqaB* (see Fig. S4). While several of these genes encode components involved in nutrient scavenging and uptake (CstA, pyruvate uptake; FruB, fructose uptake; XanP, xanthine uptake; GltS, glutamate uptake; SslE, mucin degradation), we focused our attention on the *wecA* promoter, which drives a large 12-gene operon involved in the synthesis of enterobacterial common antigen (ECA), a complex glycan polymer associated with the cell surface of *Enterobacterales* (Fig. 4a) (17).

10.1128/mbio.03246-21.4 FIG S4 ChIP-Seq analysis using HA-tagged PdeL. Chromosomally HA-tagged PdeL was used as bait with untagged PdeL as a negative control. E. coli MG1655 (CGSC7740) was used as a reference genome to align the reads from after Illumina sequencing. Blue peaks reflect PdeL binding through enrichment of the sequence coverage. Peaks height was adjusted on a linear scale to better visualize binding dynamics since the peak size was much larger for the *pdeL* promoter region than for the other peaks. The maximum enrichment is indicated for each promoter region by the number on the left of the peak. Background coverage for regions without sequence enrichment was below 500. The boxes below the peak annotate the open reading frames with the number below depicting the chromosomal position. Download FIG S4, PDF file, 0.4 MB. Copyright © 2021 Sellner et al. 2021 Sellner et al. https://creativecommons.org/licenses/by/4.0/ This content is distributed under the terms of the Creative Commons Attribution 4.0 International license.

The third gene of the *wecA* operon is *wecB* (*nfrC*), which was shown to be strictly required for N4 infection of E. coli (23). To test whether the ECA glycan polymer serves as primary receptor for phage N4, we analyzed the contribution of other *wec* genes to N4-mediated killing. Strains containing defined chromosomal deletions of *wecB* or of the entire ECA operon (Δ*eca*) were indeed resistant to N4 infection. However, deletion of the *wecA* gene alone showed no effect (Fig. 4b). This was surprising as *wecA* encodes the undecaprenyl-phosphate α-*N*-acetylglucosaminyl transferase, which catalyzes the initial step of O-antigen and ECA biogenesis (25). Importantly, expression of *wecB* alone from a plasmid fully restored N4 susceptibility of the Δ*eca* strain (Fig. 4b). This excludes the ECA as primary receptor for N4 and argues that UDP-ManNAc, the product of the WecB-mediated epimerase reaction, serves as precursor for the as-yet-uncharacterized NGR glycan polymer. In line with this, *wecB* K15A, encoding a catalytically inactive WecB variant (49), failed to restore N4 susceptibility (Fig. 4b).

Together, this implied that PdeL modulates *wecB* expression and that this may contribute to its strong protective effect against N4. To test this, we determined the exact binding site of PdeL in the *wecA* promoter region using surface plasmon resonance (SPR) and short overlapping DNA sequences covering the entire *wecA* promoter region (Fig. 4a). Strong binding of PdeL (*K_D_* 83 nM) was observed for a region overlapping two of the four transcription start sites upstream of *wecA* (50) (Fig. 4c and d). This region contains a short sequence with similarity to the PdeL binding site upstream of the *pdeL* promoter (41) and is positioned upstream of the binding site for NsrR, the only known transcription factor of the *wec* gene cluster (51) (Fig. 4a). To examine whether PdeL influences *wecA* promoter activity, we engineered a strain carrying a reporter for *wecA* transcription on the chromosome. Although *wecA* promoter activity was increased in a Δ*pdeL* mutant, it was reduced below wild-type levels in a strain expressing *pdeL* from a plasmid (Fig. 4e). Together, this suggested that PdeL is a repressor of the *wec* operon and argued that it contributes to N4 resistance by reducing c-di-GMP and as a transcriptional repressor of *wecB*, thereby limiting the availability of key components or precursors of the NGR glycan polymer.

### The N4 infection mechanism is widely conserved in pathogenic and nonpathogenic *E. coli.*

N4-like phages of the family of *Schitoviridae* were shown to infect alpha-, beta-, and gammaproteobacteria (22). To investigate the phylogenetic distribution of proteins facilitating N4 infection, we screened 1,688 bacterial genomes of the OMA database (52) for the cooccurrence of genes encoding NfrA, NfrB, WecB, and DgcJ. This analysis revealed that these genes are strongly conserved in E. coli. However, some E. coli strains (e.g., all E. coli B strains) carry insertion elements, deletions, or premature stop codons in *nfrB* or *dgcJ*, indicating that expressing these genes under laboratory conditions is associated with fitness costs.

**FIG 5:**
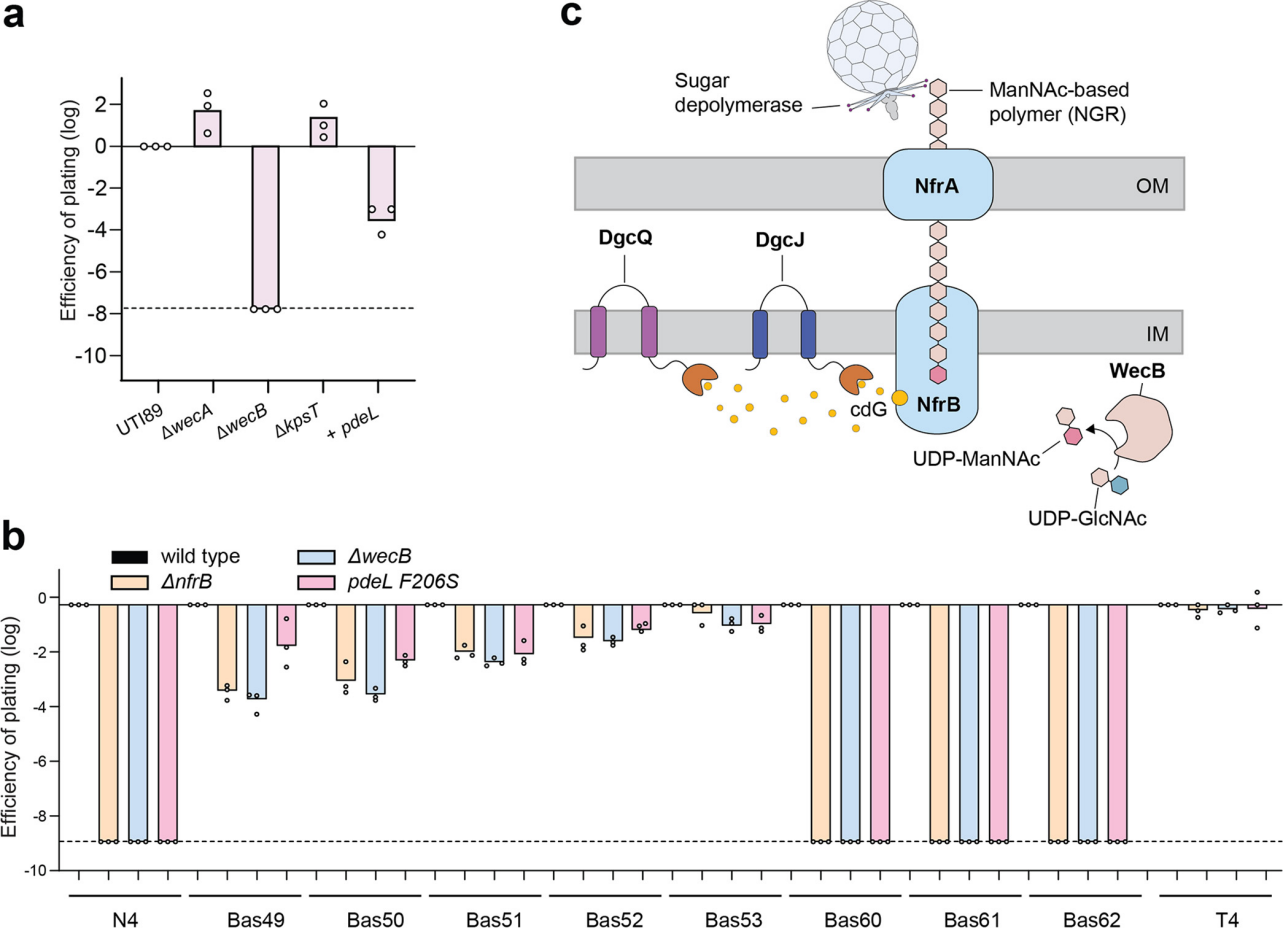
NGR is a conserved phage receptor in the pathogenic E. coli strain UTI89. (a) Phage N4 infections with the uropathogenic E. coli strain UTI89 were as described in Materials and Methods. (b) Phages from the BASEL phage collection (3) that were shown to depend on *wecB* were used to infect E. coli CGSC 6300 wild-type and selected mutant strains as indicated. (c) Model for NGR regulation and N4 adsorption (see the text for details).

Because most bacteriophages display a narrow host range (2), the remarkable conservation of N4-associated proteins in E. coli prompted us to investigate whether pathogenic E. coli strains are also susceptible to bacteriophage N4 and, if so, whether that process depends on c-di-GMP and on components of the Nfr pathway. We chose the uropathogenic E. coli K-1 strain UTI89, which in contrast to the K-12 strain MG1655 produces O-antigen and group 1 capsular polysaccharides (53), surface structures that can provide effective phage protection. As shown in Fig. 5a, UTI89 was indeed infected by phage N4, a process that was dependent on *wecB* genes but not on *wecA* (ECA and O-antigen) or *kpsT* (capsule) genes. Also, expression of *pdeL* from a plasmid resulted in complete phage protection. Together, this indicated that the primary receptor for bacteriophage N4 is widely conserved in E. coli and that surface exposure of the N4 glycan receptor follows similar regulatory logic in the pathogenic UTI89 strain as in the lab adapted, nonpathogenic E. coli K-12 MG1655.

In other Gram-negative bacteria homologs of NfrA, NfrB, WecB, and DgcJ are sporadically encoded making it difficult to assess their overall conservation and role. To identify phage representatives that exploit similar structures on the surface of their respective prey bacteria, we analyzed the available genome sequences of N4-like phages (22). While most proteins of N4-like phages are strongly conserved, tail fibers (Gp64) and tail sheaths (Gp65) are highly variable, reflecting the diversity of surface receptors of their prey (24). BLAST analysis with Gp64 and Gp65 from N4 on the family of *Schitoviridae* (22) identified a phage subgroup with conserved tail fibers and sheaths (see Table S1 in the supplemental material). This included a group of N4-like phages belonging to the subfamily of *Rothmandenesvirinae* infecting Achromobacter xylosoxidans, an opportunistic human pathogen and member of the betaproteobacteria that causes a wide range of infections, including bacteremia, meningitis, urinary tract infections, endocarditis, or pneumonia (54–57). Intriguingly, in A. xylosoxidans *nfrA*, *nfrB*, and *wecB* are cluster together with additional genes encoding putative components of exopolysaccharide biogenesis, modification, and secretion. This includes a homolog of BcsB, a component of the cellulose synthase complex (35) and homologs of periplasmic *O*-acetyltransferases involved in glycan polymer modification (58, 59) (see Fig. S5). This raises the intriguing possibility that the products of these genes are functionally linked and that NGR-like glycan polymers may serve as surface receptor for N4-like phages in a diverse range of bacterial pathogens.

10.1128/mbio.03246-21.5 FIG S5 The betaproteobacterium Achromobacter xylosoxidans harbors a gene cluster encoding homologs of *nfrA*, *nfrB*, and *wecB*. The *wecB* gene cluster of A. xylosoxidans is shown schematically with additional genes encoding a homolog of the cellulose synthase component BcsB and several homologs of periplasmic *O*-acetyltransferases (ATs). Download FIG S5, TIF file, 2.4 MB. Copyright © 2021 Sellner et al. 2021 Sellner et al. https://creativecommons.org/licenses/by/4.0/ This content is distributed under the terms of the Creative Commons Attribution 4.0 International license.

10.1128/mbio.03246-21.7 TABLE S1 Conservation of the N4 putative tail fiber (Gp64) and tail sheath (Gp65) in N4-like phages. Protein translation from the N4 phage was used as input query on the database of phages with short tails (taxid 10744). The E value is 0.0 for all listed hits, and the percent identity ranges from 99.76 to 62.44% for Gp64 and from 99.20 to 47.11% for Gp65. Download Table S1, DOCX file, 0.01 MB. Copyright © 2021 Sellner et al. 2021 Sellner et al. https://creativecommons.org/licenses/by/4.0/ This content is distributed under the terms of the Creative Commons Attribution 4.0 International license.

Recent work on the BASEL collection, a representative set of isolates from all major groups of E. coli phages, showed that phages of the *Vequintavirinae* subfamily of *Myoviridae*, their phi92-like relatives, and N4, as well as a close relative within *Enquatrovirus*, depend partially or completely on *wecB* for infectivity on the E. coli K-12 host (3). Since WecB had previously been described as being specifically required for the ECA but for no other surface glycan of E. coli (17), these results were seen as evidence for a role of ECA as primary receptor of these phages. However, our finding that at least phage N4 does not use ECA but possibly a new surface glycan, NGR, prompted us to revisit this interpretation. As expected, susceptibility of E. coli K-12 MG1655 CGSC 6300 to all tested isolates of *Vequintavirinae*, their phi92-like relatives, and *Enquatrovirus* was reduced or even abolished by a *wecB* knockout (Fig. 5b). However, none of these phages required WecA or any other part of the *wec* operon, except for *wecB* (see Fig. S6). Also, a Δ*nfrB* mutation or the *pdeL* F206S allele showed full or partial resistance, exactly phenocopying the Δ*wecB* mutant host (Fig. 5b). These results strongly suggest that none of the WecB-dependent phages use ECA as their primary receptor but that all of them instead target the NGR.

10.1128/mbio.03246-21.6 FIG S6 NGR serves as surface receptor for a range of N4-like phages infecting E. coli. CGSC 6300 wild type and mutants, as indicated by the color code, were infected with phage N4 and phages from the BASEL collection that were shown to depend on *wecB* (3). Phage infections were performed as indicated in Fig. 5b. Download FIG S6, PDF file, 0.4 MB. Copyright © 2021 Sellner et al. 2021 Sellner et al. https://creativecommons.org/licenses/by/4.0/ This content is distributed under the terms of the Creative Commons Attribution 4.0 International license.

## DISCUSSION

### NGR, an enterobacterial surface glycan commonly exploited as a phage receptor.

Our results show that N4 and other phages previously thought to target the ECA to infect E. coli use a novel surface-associated polysaccharide that we call NGR (N4 glycan receptor) as their primary receptor. We suggest that biosynthesis and export of NGR depend on a conserved machinery including WecB, as well as on NfrB and NfrA, which share strong homology to known polysaccharide export systems (Fig. 5c). The conservation of this *bona fide* NGR biosynthesis machinery may well explain the remarkably broad host recognition among phages targeting this surface structure among enterobacteria (3). This raises the question why ECA does not seem to be used by phages to a similar extent because, to the best of our knowledge, there is no remaining bacteriophage thought to target this surface glycan. One possibility could be differences in expression or insufficient size of the ECA to be useful as a receptor and, therefore, the availability of these glycans as a receptor in the true habitats of enterobacteria. We anticipate that future studies exploring the biology of these elusive polysaccharides might also help to understand their very different use as bacteriophage receptors.

Based on the observation that the sugar epimerase WecB is essential for phage infection, we propose that NGR is a polymer containing the monosaccharide *N*-acetylmannosamine (ManNAc) that is produced by this enzyme. This is in line with *wecB* being the only gene of the ECA cluster required for N4 infection and the only EPS biosynthesis gene ever identified in genetic screens for N4 resistance (10, 23). Uncovering the molecular identity of NGR, its overall conservation, and the details of its biosynthesis and export will require further studies. Notably, ManNAc-based EPS components are widely used by other bacteria. For instance, Neisseria meningitidis serogroup A produces a poly-ManNAc capsule, which serves as a primary virulence factor to promote host colonization and serum resistance (60, 61). Similarly, neuroinvasive E. coli K1 (62) requires ManNAc as a building block for sialic acid, the repeat unit of their capsule (63). In E. coli K1 strains, UDP-GlcNAc is converted to ManNAc by the epimerase NeuC, but this mechanism is catalytically distinct from WecB (64). The observation that deletion of the *wecB* gene in the K1 strain UTI89 leads to complete N4 resistance argues that the substrate for the polymerization of NGR is provided by WecB and not by NeuC.

The specific role of NGR remains unclear. Its widespread distribution and conserved surface exposure despite of potentially strong selection by phage predators argues that it has a vital role in E. coli and *Enterobacterales*. Similarly, the functional significance of ECA has remained enigmatic. Mutants lacking ECA are more sensitive to different forms of stress, have increased outer membrane permeability, and show reduced virulence (17). It is possible that NGR has similar protective roles in E. coli and its relatives or is involved in bacterium-host interaction. A specific role for NGR in the host environment is supported by the observation that *dgcJ* expression limits NGR biogenesis and N4 infection at 30°C but increases strongly when cells are shifted to 37°C. NGR expression in the host could protect from phagocytosis, similar to capsules, or through masking patterns on the bacterial surface to avoid recognition from the immune system. Alternatively, NGR may have evolved in nonpathogenic members of the microbiota to avoid encounters by the immune system. This would be in line with strong structural conservation of the NGR surface glycan because the structure would be defined through receptor binding to the eukaryotic cells in the gut.

### NGR biogenesis is controlled by local c-di-GMP signaling.

Based on its strong homology to membrane-embedded glycosyltransferases, we propose that NfrB is responsible for NGR polymerization (Fig. 5c). We propose that NfrB activity is regulated by c-di-GMP binding to the MshEN-like domain, which is positioned adjacent to the glycosyltransferase domain. This is reminiscent of the E. coli cellulose synthase BcsA, in which the catalytically autoinhibited state is released by binding of c-di-GMP to an associated PilZ domain (65, 66). Importantly, BcsA is specifically regulated by the diguanylate cyclase DgcC and the phosphodiesterase PdeK, which interact directly with the cellulose synthase complex. It was proposed that this arrangement provides a target-specific pool of c-di-GMP to locally boost the activity of BcsA, thereby sequestering the regulation of the cellulose synthase complex from global fluctuations of the second messenger (67). Our findings indicate that NfrB activity may be controlled in a similar manner. Not only did large changes of the global c-di-GMP concentration not affect N4 susceptibility (despite of their strong effect on other c-di-GMP-dependent cellular functions like motility), but also, N4 infection was critically dependent on *dgcJ* (but none of the other *dcg* genes) even in the hypermotile strain CGSC 7740, which harbors low levels of c-di-GMP. Thus, DgcJ likely acts as “local pacemaker” (68) to specifically regulate NGR biogenesis in response to environmental cues (Fig. 5c). This idea is supported by a parallel study showing that c-di-GMP indeed binds to the MshEN-like domain of NfrB and also demonstrating a direct interaction between NfrB and DgcJ (27).

It is possible that E. coli uncouples the regulation of NGR biogenesis from global cellular changes of c-di-GMP to avoid directly linking the exposure of this surface glycan to general lifestyle changes mediated by c-di-GMP (69, 70). This would not only increase the precision of NfrB regulation, but would limit NGR surface exposure to appropriate and highly distinct environmental conditions and thereby strongly reduce the selective pressure exerted by a diverse range of bacteriophages that exploit strongly conserved surface structures as receptors. To better understand this “Achilles’ heel” strategy, it will be interesting to identify the environmental cues that activate DgcJ and stimulate NGR surface exposure. The periplasmic dCache domain of DgcJ shows homology to chemosensory domains of P. aeruginosa, which bind amino acids and autoinducer-2 (47, 71). DgcJ may well respond to similar nutritional or cell density-related cues, which again raises the question of NGR functionality and why E. coli would need to expose the NGR glycan under such highly specific conditions. The role and specificity of DgcQ is less clear since it seems to have an auxiliary function in activating NGR biogenesis in response to the presence of arginine in the growth medium. Although we cannot exclude that DgcQ locally cooperates with DgcJ, we find it more plausible that it supports NGR biogenesis by increasing the global c-di-GMP pool of E. coli.

While the specific role of DgcJ can be explained by its spatial coupling to the NGR biosynthesis machinery (27), the highly effective role of PdeL in protecting E. coli from N4 lysis is likely due to its dual function as a phosphodiesterase and as a transcription factor (41). Although the catalytic activity of PdeL is clearly important for N4 protection, PdeL is a transcription factor and thus unlikely to act in a spatially confined compartment. Rather, we speculate that PdeL interferes with NGR biogenesis by repressing *wecB* transcription and, consequently, by limiting WecB-dependent ManNAc supply for NGR biosynthesis. The prominent role of PdeL in regulating NGR and N4 phage sensitivity may also relate to its bimodal expression that was shown to generate distinct E. coli subpopulations with high and low levels of c-di-GMP, respectively (72). We therefore speculate that PdeL bimodality may be part of a bet-hedging mechanism that specifically protects a fraction of clonal bacterial populations from phage predation by preventing the production of NGR during conditions that would induce NGR biosynthesis.

## MATERIALS AND METHODS

### Bacterial strains and growth conditions.

The bacterial strains and plasmids used in this study are listed in Table S2. E. coli K-12 MG1655 wild-type strains were ordered from the Coli Genetic Stock Center and indicated with their accession number. Strains were grown in glass culture tubes with agitation at 170 rpm on 37°C or 30°C. When needed antibiotics were present at the following concentrations: 50 μg/mL kanamycin and 30 μg/mL for low- or single-copy plasmids.

### Culture media and solutions.

Lysogeny broth (LB) was prepared by dissolving 10 g/L tryptone, 5 g/L yeast extract, and 10 g/L NaCl in Milli-Q H_2_O. LB agar plates were prepared by supplementing LB medium with 1.5% (wt/vol) agar (AppliChem). Top agar was prepared by supplementing LB containing 0.4% agarose with 20 mM MgSO_4_ and 5 mM CaCl_2_ and stored at 60°C for up to 4 weeks.

MOPS defined medium was prepared as previously described (73) in Milli-Q H_2_O. Phosphate-buffered saline (PBS) was prepared as a solution containing 1.44 g/L Na_2_HPO_4_, 0.24 g/L KH_2_PO_4_, 0.2 g/L KCl, and 8 g/L NaCl in Milli-Q H_2_O adjusted to a pH of 7.4 using 10 M NaOH. SM buffer was prepared as a solution of 0.1 M NaCl, 10 mM MgSO_4_, and 0.05 M Tris-HCl (pH 7.5). PdeL purification buffer contains 200 mM NaCl, 5 mM MgCl_2_, 5 mM dithiothreitol, and 20 mM Tris-HCl (pH 8.0). DNA hybridization buffer contains 150 mM NaCl and 15 mM trisodium citrate in Milli-Q H_2_O adjusted to a pH of 7.

### Chromosomal gene deletions and modifications.

Gene deletions were carried out either by transduction from the Keio collection (74) or using λ-red homologous recombination using pKD46, as described previously (75). Selection markers were removed by site-specific recombination using pCP20 (75).

### Plasmid construction.

Plasmids were constructed either using classic restriction-based molecular cloning or Gibson assembly (76). Plasmids were transformed into E. coli DH5α and purified using a GenElute miniprep kit from Sigma-Aldrich.

### Microscopy.

Bacteria were grown to exponential phase in LB at 37°C and transferred on a 0.75-mm-thick agarose pad containing PBS and 1% agarose. Images were acquired using an Eclipse Ti2 inverted microscope (Nikon) equipped with an ORCA-Flash4.0 CMOS camera C11440-22C (Hamamatsu), and an CFI PlanApo DM 100× Lamda Oil/1.45/0.13 objective (Nikon). Bright-field images were illuminated using the High-Power LED-100 Illumination system (Nikon) at a 50-ms exposure time. Fluorescence of GFPmut2 was acquired at 470/24 nm with a 100-ms exposure time. The open-source software Oufti (77) was used for automatic cell detection and WHISIT (78) to quantify the fluorescence intensity.

### Swimming assay.

Swimming assays were performed as described previously (79) with small modifications. In brief, 2.5 μL of E. coli overnight culture was transferred on a plate containing 10 g/L tryptone, 5 g/L NaCl, and 0.3% agar (AppliChem) and incubated at 37°C. After 7 to 15 h, the swimming area was measured, and the relative swimming distance was calculated using E. coli wild type as a reference.

### Phage lysate preparation.

P1 phage lysate preparation and transduction were performed as described previously (80). N4 and T5 phage lysates were prepared as described previously (3) and stored in SM buffer.

### Phage infection assay.

Phage infections were adapted from (3). In brief, 100 μL of E. coli overnight culture was mixed with 3 mL of top agar and poured on an LB agar plate prewarmed to 60°C. The top agar solidifies after 15 min at room temperature, allowing to spot 2.5 μL of a 10-fold serial diluted phage solution on the double-agar overlay plate. After the spots were dried, the plate was incubated at 37°C. PFU were counted after 12 to 18 h to calculate the efficiency of plating (EOP).

### Protein purification.

PdeL-6×His was expressed from pET28a in BL21 cells grown at 22°C for 5 h in 2 L of LB. Cells were harvested by centrifugation at 6,000 × *g* for 30 min at 4°C. The cell pellet was resuspended in 10 mL of PdeL purification buffer and one tablet of Complete mini-EDTA-free protease inhibitor (Roche), and a spatula tip of DNase I (AppliChem) was added to the cell suspension. Cells were lysed by three passages of French press, and the lysate was cleared at 100,000 × *g* for 1 h in an ultracentrifuge at 4°C. The supernatant was added to 2 mL of Protino Ni-NTA agarose slurry in a 15-mL Falcon tube and rotated slowly at 4°C for 30 min to allow for binding. The slurry was then filled in a gravity-flow column, washed with 10 mL of PdeL purification buffer, and then with 40 mL of the same buffer supplemented with 40 mM imidazole. Proteins were eluted with 10 mL of PdeL purification buffer supplemented with 500 mM imidazole. The eluted protein was then loaded onto a HiLoad 16/600 Superdex 200-pg size exclusion column for fractionation using 140 mL of PdeL purification buffer. The protein concentration of the appropriate fractions was determined by photo-spectrometric absorption at 280 nm and used fresh or stored at −80°C.

### SPR measurements.

The affinity of PdeL to DNA sequences was determined by SPR measurements using the ReDCaT method (81). In brief, hybridized biotinylated DNA was immobilized on a streptavidin-coated SPR chip (Cytiva). Experiments were performed at 4°C in a GE Biacore T200 SPR instrument using a flow rate of 10 μL/min. Washing and regeneration of the chip was performed using 1 M NaCl and subsequently 50 mM NaOH at a 10-μL/min flow rate. PdeL buffer was supplemented with 0.1 mg/mL bovine serum albumin and 20 ng/μL salmon sperm DNA to reduce unspecific interactions. The *K_D_* was determined using a Michaelis-Menten kinetic fitting model.

10.1128/mbio.03246-21.8 TABLE S2 Plasmids, strains, and primers used in this study. Download Table S2, DOCX file, 0.02 MB. Copyright © 2021 Sellner et al. 2021 Sellner et al. https://creativecommons.org/licenses/by/4.0/ This content is distributed under the terms of the Creative Commons Attribution 4.0 International license.
