# Supplementary figures and images for: A New Sugar for an Old Phage: a c-di-GMP-Dependent Polysaccharide Pathway Sensitizes Escherichia coli for Bacteriophage Infection
Source: mBio. 2021 Dec 14;12(6):e03246-21. doi: 10.1128/mbio.03246-21 (PMC8669472; doi:10.1128/mbio.03246-21)

**NfrA**

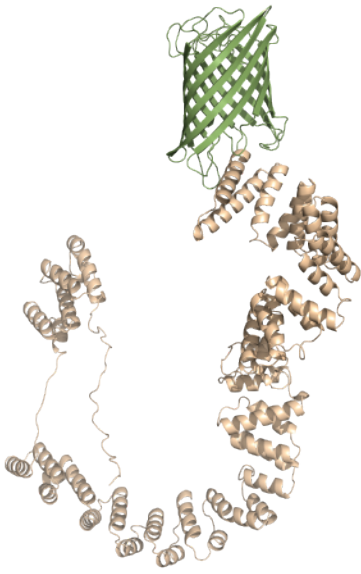

**BcsC**

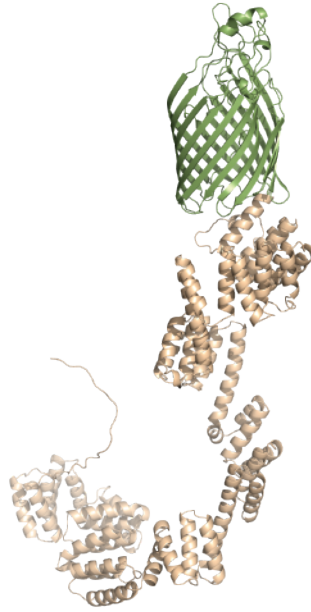

**PgaA**

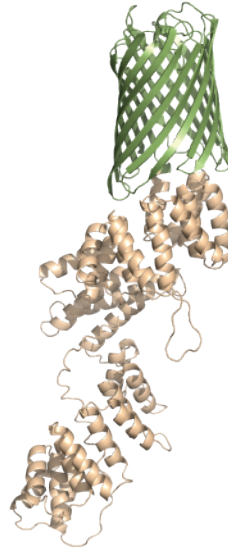

Supplement: FIG S1 [file mbio.03246-21-sf001.pdf]

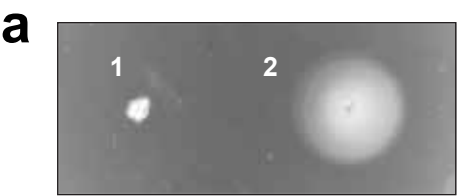

**b**

|                                 | 1 | 2 | 3 | 4 | 5 | 6 | 7 | 8 | 9 |
|---------------------------------|---|---|---|---|---|---|---|---|---|
| IS1 in <i>P<sub>flhDC</sub></i> | - | + | - | + | + | + | - | - | - |
| IS1 in <i>crl</i>               | - | + | + | - | + | - | - | + | - |
| IS1 in <i>dgcJ</i>              | - | + | + | + | - | - | + | - | - |

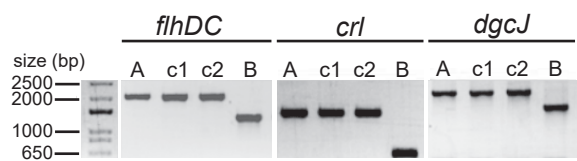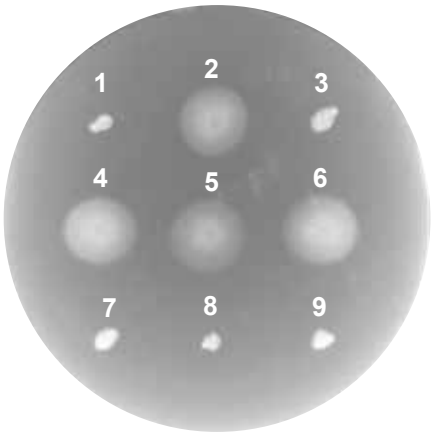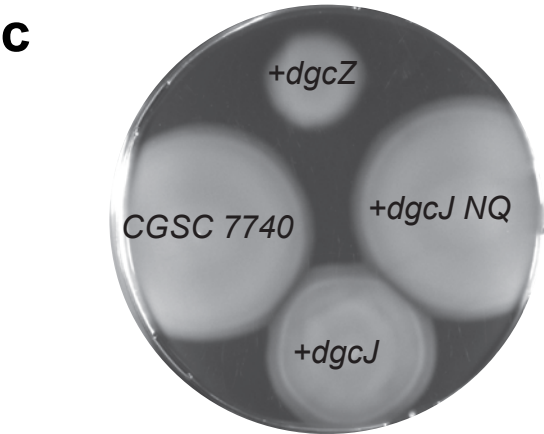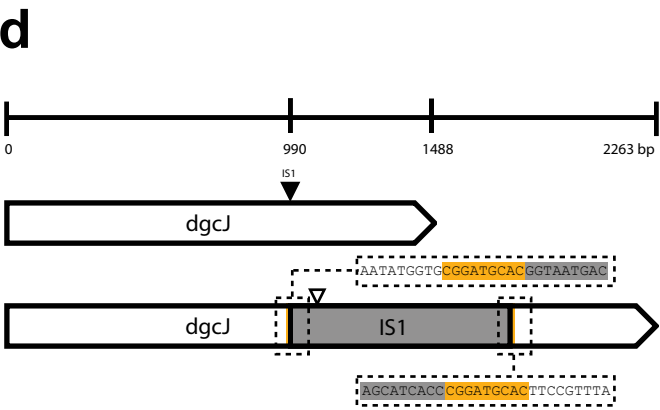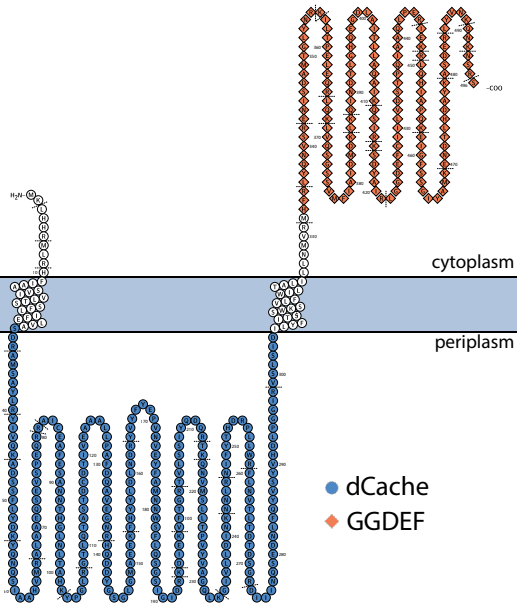

Supplement: FIG S2 [file mbio.03246-21-sf002.pdf]

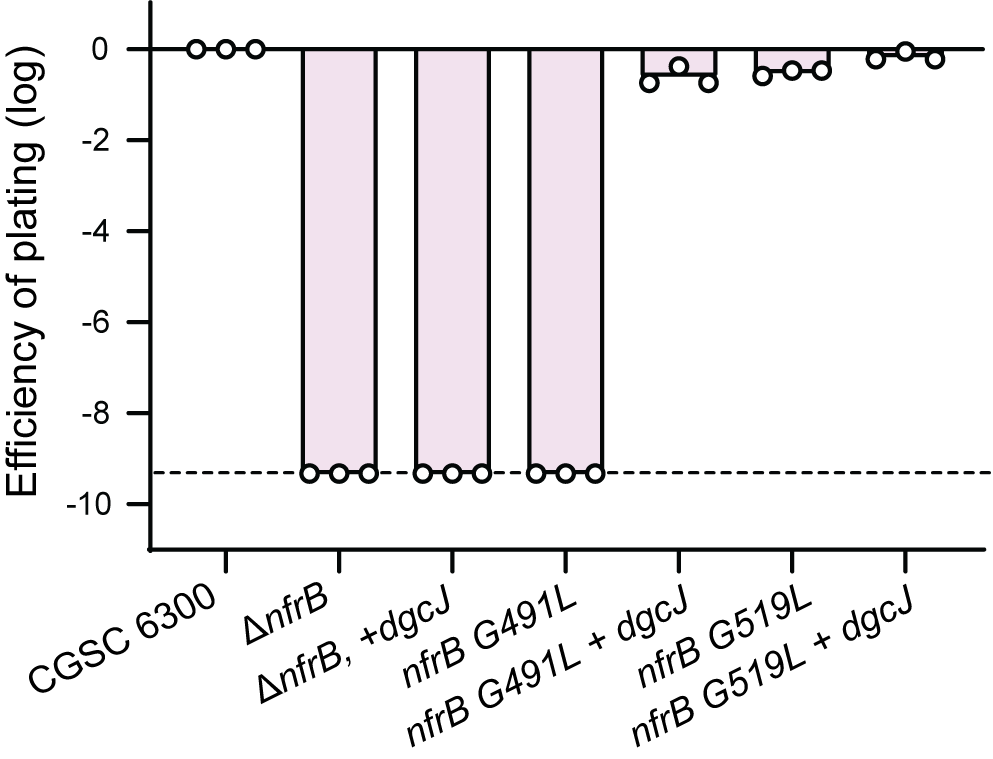

Supplement: FIG S3 [file mbio.03246-21-sf003.tif]

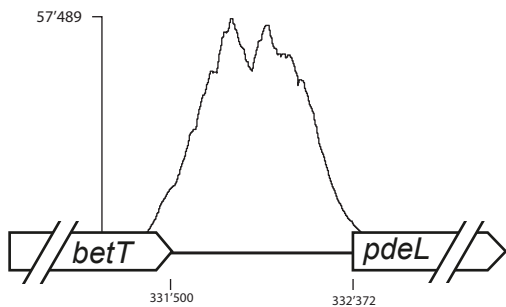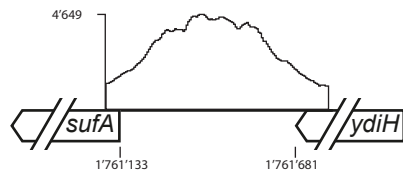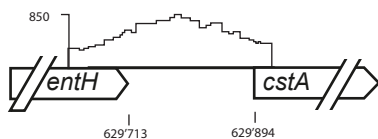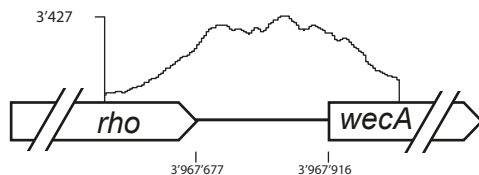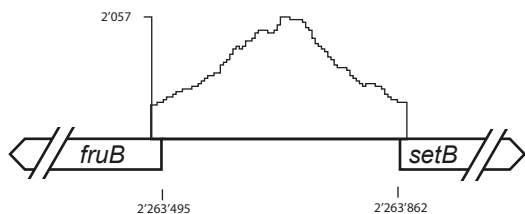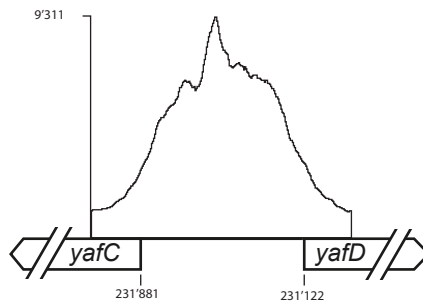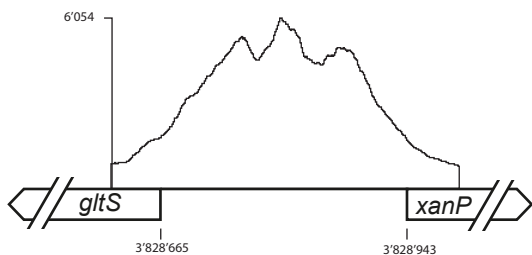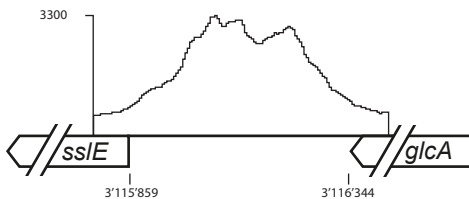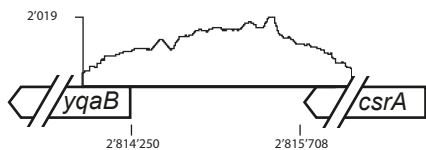

Supplement: FIG S4 [file mbio.03246-21-sf004.pdf]

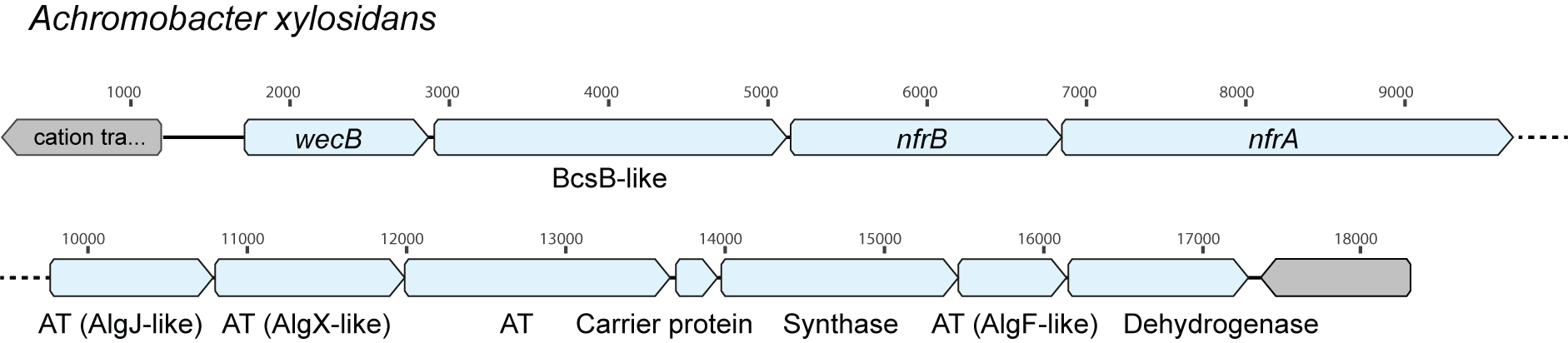

Supplement: FIG S5 [file mbio.03246-21-sf005.tif]

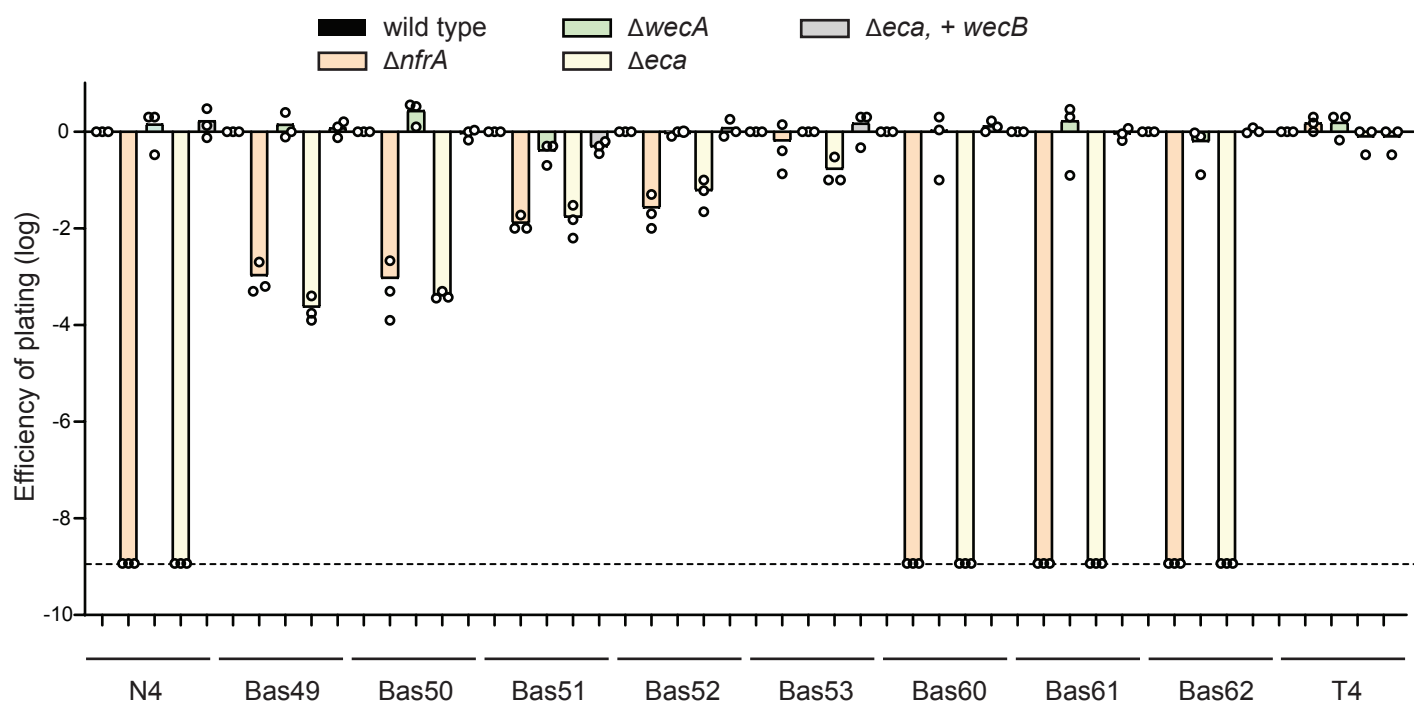

Supplement: FIG S6 [file mbio.03246-21-sf006.pdf]
