# Supplementary material for: A New Sugar for an Old Phage: a c-di-GMP-Dependent Polysaccharide Pathway Sensitizes Escherichia coli for Bacteriophage Infection
Source: mBio. 2021 Dec 14;12(6):e03246-21. doi: 10.1128/mbio.03246-21 (PMC8669472; doi:10.1128/mbio.03246-21)
Supplement: TABLE S1 [file mbio.03246-21-st001.docx]

**Table S1**

| Phage | conserved | | Host |
| --- | --- | --- | --- |
|  | Gp64 | Gp65 |  |
| VEc25 | yes | yes | *Escherichia coli* |
| vB_EcoP_3HA13 | no | yes | *Escherichia coli* O111 |
| OLB145 | no | yes | *Escherichia coli* |
| PMBT57 | yes | yes | *Escherichia coli* O111:H- |
| vB_AxyP_19-31_Axy04 | yes | yes | *Achromobacter xylosoxidans* |
| vB_AxyP_19-31_Axy10 | yes | yes | *Achromobacter xylosoxidans* |
| vB_AxyP_19-31_Axy11 | yes | yes | *Achromobacter xylosoxidans* |
| vB_AxyP_19-31_Axy12 | yes | yes | *Achromobacter xylosoxidans* |
| vB_AxyP_19-31_Axy13 | no | yes | *Achromobacter xylosoxidans* |
| vB_AxyP_19-31_Axy22 | no | yes | *Achromobacter xylosoxidans* |
| vB_AxyP_19-31_Axy24 | yes | yes | *Achromobacter xylosoxidans* |
| phiAxp-3 | yes | yes | *Achromobacter xylosoxidans* |
| Inbricus | yes | yes | *Pseudomonas syringae* pv. avii |
| RG-2014 | yes | yes | *Delftia tsuruhatensis* ARB-1 |
