# Supplementary material for: A New Sugar for an Old Phage: a c-di-GMP-Dependent Polysaccharide Pathway Sensitizes Escherichia coli for Bacteriophage Infection
Source: mBio. 2021 Dec 14;12(6):e03246-21. doi: 10.1128/mbio.03246-21 (PMC8669472; doi:10.1128/mbio.03246-21)
Supplement: TABLE S2 [file mbio.03246-21-st002.docx]

## Table S2: Plasmids, strains and primers

| Plasmid | Description | Source/Reference |
| --- | --- | --- |
| pKD46 | λ RED^+^ (*amp*) | Datsenko et al. |
| pCP20 | FLP^+^ (*amp*) | Cherepanov et al. |
| pAR78 | P_lac_-RBS_synth._-*pdeL(E141A)-3xflag* in pNDM220 (*amp*) | this study |
| pAR81 | P_lac_-RBS_synth._-*pdeH-3xflag* in pNDM220 (*amp*) | this study |
| pAR88 | P_lac_-RBS_synth._-dgcZ*-3xflag* in pNDM220 (*amp*) | this study |
| pAR172 | 3xFlag-3xSTOP-RBS-mCherry-3xSTOP-mCherry::cat in pUC19 | this study |
| pAR281 | kan cassette with FRT sites in pNDM220 | this study |
| pAR297 | P_dgcJ_-GFPmut2 in pUA66 (*kan*) | this study |
| pAR314 | P_lac_-RBS-dgcJ in pNDM220 (*amp*) | this study |
| pAR314 | P_lac_-RBS-dgcJ in pNDM220 (*amp*) | this study |
| pAR315 | P_lac_-RBS-dgcJ (GGNQF) in pNDM220 (*amp*) | this study |
| pAR321 | P_lac_-RBS-wecB in pNDM220 (*amp*) | this study |
| pAR354 | Plac-RBS-wecB (K15A) in pNDM220 (amp) | this study |

| **Strain** | | **Genotype** | **Source/Reference** | |
| --- | --- | --- | --- | --- |
| CGSC6300 | | *E. coli K-12* | Coli Genetic Stock Center (Yale) | |
| CGSC7740 | | *E. coli K-12, P_flhD_::IS1, dgcJ::IS1* | Coli Genetic Stock Center (Yale) | |
| AB3851 | | *UTI89 (O18:K1:H7)* | (Chen et al., 2006) | |
| AB607 | | *P_flhD_::IS1, dgcJ::IS1, ΔpdeH* | (Boehm et al., 2010) | |
| AB3316 | | *pdeL-HA-kan::frt* | this study | |
| AB3567 | | *PflhD::IS1, dgcJ::IS1, crl::IS1 wecA-3xFlag-3xSTOP-RBS-mCherry-3xSTOP-RBS-mCherry-cat::frt* | this study | |
| AB3581 | | *PflhD::IS1, dgcJ::IS1, crl::IS1 ΔpdeL::frt, wecA-3xFlag-3xSTOP-RBS-mCherry-3xSTOP-RBS-mCherry-kan::frt* | this study | |
| AB3864 | | *P_flhD_::IS1, Δ(dgcJ::IS1)::dgcJ+, crl::IS1* | this study | |
| AB3870 | | *Δ(P_flhD_::IS1)::P_flhD_+, dgcJ::IS1, crl::IS1* | this study | |
| AB3882 | | *PflhD::IS1, dgcJ::IS1, Δ(crl::IS1)::crl+* | this study | |
| AB3897 | | *Δ(P_flhD_::IS1)::P_flhD_+, , Δ(dgcJ::IS1)::dgcJ+, crl::IS1* | this study | |
| AB4423 | | *ΔwecA::frt* | this study | |
| AB4435 | | *ΔnfrA::frt* | this study | |
| AB4436 | | *ΔnfrB::frt* | this study | |
| AB4456 | | *ΔdgcJ::frt* | this study | |
| AB4463 | | *UTI89 (O18:K1:H7), ΔkpsT::kan* | this study | |
| AB4465 | | *ΔwecA-rffM::frt* | this study | |
| AB4477 | | *UTI89 (O18:K1:H7), ΔwecA::kan* | this study | |
| AB4478 | | *UTI89 (O18:K1:H7), ΔwecB::kan* | this study | |
| AB4487 | | *UTI89 (O18:K1:H7), pdeL(D295N)-kan::frt* | this study | |
| AB4492 | | *ΔpdeH* | this study | |
| AB4518 | | *pdeL::frt* | this study | |
| AB4523 | | *ΔwecB::frt* | this study | |
| AB4558 | | *dgcJ(Y168F)-kan::frt* | this study | |
| AB4559 | | *dgcJ(Y210F)-kan::frt* | this study | |
| AB4560 | | *dgcJ(D212N)-kan::frt* | this study | |
| AB4561 | | *dgcJ(D239N)-kan::frt* | this study | |
| AB4562 | | *∆dgcJ::Frt, ∆dgcQ::Frt* | this study | |
| AB4563 | | *ΔdgcQ::frt* | this study | |
| AB4595 | | *ΔdgcE::frt* | this study | |
| AB4646 | | *ΔdgcC::frt* | this study | |
| AB4647 | | *ΔdgcF::frt* | this study | |
| AB4648 | | *ΔdgcI::frt* | this study | |
| AB4649 | | *ΔdgcM::frt* | this study | |
| AB4650 | | *ΔdgcN::frt* | this study | |
| AB4651 | | *ΔdgcO::frt* | this study | |
| AB4652 | | *ΔdgcP::frt* | this study | |
| AB4653 | | *ΔdgcT::frt* | this study | |
| AB4654 | | *ΔdgcZ::frt* | this study | |
| AB4690 | | *pdeL(F206S)-3xFlag-kan::frt* | this study | |
|  | |  |  | |
| **Primers** | |  |  | |
| **Primer** | **Sequence** | | **Restriction Site** | **Used for plasmid or strain** |
| 6805 | GAAAAAAAGGTACCATAGGAGGAACAATTTTATGAATTCATGTGATTTTCGTG | | KpnI | pAR71/pAR78 |
| 6806 | GAAAAAAAGGTACCTTATTTATCGTCGTCATCTTTGTAG | | KpnI | pAR71/pAR78 |
| 7323 | GAAAAAAAGGATCCATAGGAGGAACAATTTTATGATAAGGCAGGTTATCCAGC | | BamHI/XhoI | pAR81 |
| 7324 | GAAAAAAACTCGAGTTATTTATCGTCGTCATCTTTGTAGTCGATATCATGATCTTTATAATCACCGTCATGGTCTTTGTAGTCGAATAGCGCCAGAACCGCCGTATTC | | BamHI/XhoI | pAR81 |
| 7619 | GAAAAAAAGGTACCATAGGAGGAACAATTTTATGATCAAGAAGACAACGGAAATTG | | KpnI | pAR88 |
| 7620 | GAAAAAAAGGTACCTTATTTATCGTCGTCATCTTTGTAG | | KpnI | pAR88 |
| 9604 | GAAAAAAAGAATTCTTCGACTACAAAGACCATGACGGTGATTATAAAGATCATGATATCGACTACAAAGATGACGACGATAAATAATAATAATAAATAGGAGGAACAAT | | BamHI/EcoRI | pAR172 |
| 8312 | GAAAAAAAGGATCCGAATATCCTCCTTAGTTCCTATTCCG | | BamHI/EcoRI | pAR172 |
| 13778 | CCGCTCGAGGACACGCGTAATATCTCGC | | XhoI/BamHI | pAR297 |
| 13779 | CGGGATCCCTCTGTCGCTGGCGACAAGTTC | | XhoI/BamHI | pAR297 |
| 15080 | TAAAAGGTACCGGCCGCAATCGAACTTTATCTGG | | KpnI/XhoI | pAR314 |
| 15081 | TAAAACTCGAGTCATGAACGGCTGTTTTTGTTCTGC | | KpnI/XhoI | pAR314 |
| 15152 | GATTATGCCATCCGACTCGGTGGCAATCAATTCTGCATCATTCTTGTCGATTCG | | | pAR315 |
| 15081 | TAAAACTCGAGTCATGAACGGCTGTTTTTGTTCTGC | | KpnI/XhoI | pAR315 |
| 15153 | GGCTGTTTTTGTTCTGCTTATTGACA | | | pAR315 |
| 15080 | TAAAAGGTACCGGCCGCAATCGAACTTTATCTGG | | KpnI/XhoI | pAR315 |
| 15423 | TAAAGGTACCGCGCTCGCCGCTTATTCGAAGAGAA | | KpnI/XhoI | pAR321 |
| 15424 | TAAACTCGAGTCATAGTGATATCCGATTATTT | | KpnI/XhoI | pAR321 |
| 16023 | AAGGGAATAAGGGCGACACG | | KpnI/XhoI | pAR354 |
| 16024 | CATGCACCAACGGCGCCATTGCGATGGCTTCCGGGCGC | | | pAR354 |
| 16025 | GCGCCCGGAAGCCATCGCAATGGCGCCGTTGGTGCATG | | | pAR354 |
| 16026 | TTTCCCAGTCACGACGTTGT | | KpnI/XhoI | pAR354 |
| 11203 | TGGTGGCAGCCCCAATTTAACCAAATAATTCGACTACAAAGACCATGACGGTGATTATAAAGATCATG | | | AB3567 |
| 11204 | CGGCCGGTTTCCCAGGCATTGGTTGTGTCATCACATCCTCATGAATATCCTCCTTAGTTCCTATTCC | | | AB3567 |
| 13973 | GCTAAATTTTGCCAATTTGGTAAAACAGTTGCATCACAACAGGAGATAGCATCAGAAGAACTCGTCAAGAAG | | | AB3882 |
| 13974 | GAATATTAATAATGATGATATTGTCGCGGTTGGCATGACGTATCAGTTTTAATGGGATATTATCGTGAGGATG | | | AB3882 |
| 13975 | GCTAAATTTTGCCAATTTGGTAAAACAGTTGCATCACAACAGGAGATAGCA | | | AB3882 |
| 13976 | GAATATTAATAATGATGATATTGTCGCGGTTGGCATGACGTATCAGTTTTAATG | | | AB3882 |
| 12820 | GATTTTCAATAATGCGTGATGCAGATCACACAAAACACTCAATTACTTAACTCAGAAGAACTCGTCAAGAAG | | | AB3870 |
| 12821 | CTTTGTATTTAATTAGTTTGTTGTGCGGTAAGTGTCTGTTTAAAAATAGCGGATATTATCGTGAGGATG | | | AB3870 |
| 12822 | GAAGTGACAAACCAGTTGATTG | | | AB3870 |
| 12823 | CTTTGCGTTTCTTCATGCATC | | | AB3870 |
| 15081 | TAAAACTCGAGTCATGAACGGCTGTTTTTGTTCTGC | | | AB4558 |
| 15958 | GATCTCAATGACCGCTATGTCTATTTTTTCGAGCCGGTTAATGTTGAATACTTTG | | | AB4558 |
| 15080 | TAAAAGGTACCGGCCGCAATCGAACTTTATCTGG | | | AB4558 |
| 15957 | CAAAGTATTCAACATTAACCGGCTCGAAAAAATAGACATAGCGGTCATTGAGATC | | | AB4558 |
| 15081 | TAAAACTCGAGTCATGAACGGCTGTTTTTGTTCTGC | | | AB4559 |
| 15962 | CTGTTTAGTACGCTGATCCTGAAAAATGCTCGACAATACGGTAC | | | AB4559 |
| 15080 | TAAAAGGTACCGGCCGCAATCGAACTTTATCTGG | | | AB4559 |
| 15963 | GTACCGTATTGTCGAGCATTTTTCAGGATCAGCGTACTAAACAG | | | AB4559 |
| 15081 | TAAAACTCGAGTCATGAACGGCTGTTTTTGTTCTGC | | | AB4560 |
| 15985 | CATCACGTTCTGTTTAGTACGCTGATTCTGGTAAATGCTCGACAATACGG | | | AB4560 |
| 15080 | TAAAAGGTACCGGCCGCAATCGAACTTTATCTGG | | | AB4560 |
| 15986 | CCGTATTGTCGAGCATTTACCAGAATCAGCGTACTAAACAGAACGTGATG | | | AB4560 |
| 15081 | TAAAACTCGAGTCATGAACGGCTGTTTTTGTTCTGC | | | AB4561 |
| 15271 | GCTAAAAGGGATTGTGCTGCTGAATATTAACAAAAACAATCTGCGGAATATC | | | AB4561 |
| 15080 | TAAAAGGTACCGGCCGCAATCGAACTTTATCTGG | | | AB4561 |
| 15272 | GATATTCCGCAGATTGTTTTTGTTAATATTCAGCAGCACAATCCCTTTTAGC | | | AB4561 |
| 15552 | CGAAATGATGAAACGCTCATAAATTTGTCTTATGCCAAAAACGCCACGTGTTTAGATTGTGTAGGCTGGAGCTGCTT | | | AB4465 |
| 15553 | GTAATAAAAAAGCAGACAGGCGACGGAGTGACCACTCCGTCGCTTTACAAAGAGAGGAAAAGAATTCGAATATCCTCCTTAGTT | | | AB4465 |
| 15684 | GGTCTTCGTGGTTATACTTCTGCTAATAATTTTCTCTGAGAGCATGCATTATTGTGTAGGCTGGAGCTGCTTCG | | | AB4477 |
| 15685 | AGCGTCTTCGGCCGGTTTCCCAGGCATTGGTTGTGTCATCACATCCTCATGAATTCGAATATCCTCCTTAGTTCC | | | AB4477 |
| 15661 | CAACACTGCTGCGGTGAGCGCAAAGGCGCTCGCCGCTTATTCGAAGAGAATCGATGATTGTGTAGGCTGGAGCTGC | | | AB4478 |
| 15662 | TAGGCAGCCCGATGTAACCCAGTCCGATAACAGAAATGGTCGCAAAACTCATGAATTCGAATATCCTCCTTAGTTCC | | | AB4478 |
| 15535 | CTTTTTACTCATGATATGTCTCAGTTAAATATTTCCAAGGCATAATATTGGAATGTAACCAGATTGTGTAGGCTGGAGCTGCTTCGAA | | | AB4463 |
| 15536 | GGTCAAACCGTCTGAGTAAATTTTATCCAGTTACAAACAAGTATTACCTCCAGTGTAGAATTCGAATATCCTCCTTAGTTCCTA | | | AB4463 |
| 5660 | GATGTTCCCAGCGGACAAGCACCGCACAGCCCGTCAGTACGCCAGTC | | | AB2715 |
| 5661 | GTGCTTGTCCGCTGGGAACATC | | | AB2715 |
